# What Was It?

**Published:** 1874-05

**Authors:** John W. Booth

**Affiliations:** Tally-Ho, N. C.


### WHAT WAS IT?

BY JOHN W. BOOTH, M.D., OF TALLY-HO, N. C.

Dr. Kirkpatrick, in the RECORD for January, 1873, gives a short account of a girl, that went into the creek as she began to menstruate, and stopped the flow which was followed by menorrhagia, etc., but unfortunately he does not give sufficient account of his case, to assist us in treating just such an one or even recognizing it to be the same if we had it. Would not a physical examination have set him right and made him able to decide what it was? The physician is often led to make a mistake, which he can not afford to do, or to let his poor patient suffer unnecessarily, by neglect of these physical examinations.

One practitioner of my acquaintance attended a young married lady, who had never menstruated. She complained of nothing else. He gave her emmenagogues, etc., ad infinitum and wondered what the reason was, but to no purpose. The second doctor that was consulted, by means of his probe in the bladder, and his finger in the rectum, etc., diagnosed the *absence of a uturus* and thereby won laurels for himself, that the first might have worn.

Dr. K's case reminds me of one, that I treated in 1869, in a married lady. The history of her case is as follows: She menstruated early and it was normal and painless. But at the age of 14 she was walking some distance to school, and in autumn, the dews were heavy and the weeds were high on her path, so that she got wet up to her waist every morning, and had to sit with her wet clothes on until they dried. The flow commenced at one time, and was checked by this cause. For a long time afterwards, as I was told by the physician that attended her, she never had any flow, except under the influence of aloetic purges, although she did not suffer any pain or inconvenience from this state of things, and was unusually robust and athletic girl, weighing not much less than two hundred pounds, and being fond of pedestrian exercise.

She married at about thirty, and after her marriage had no trouble sufficient to make her consult a physician until 1863, some three or four years after her marriage, when she had metrorrhagia for which she took physic with some benefit.

When she came under my care in 1869, she had for some time had no flow during six months at a time, and then had a continuous metrorrhagia more or less for the same length of time.

She had remained sterile and is so yet. She suffered a good deal of pain, in the left hypochondriac and the lumbar region, but had the appearance of a person in perfect health, had a first-rate appetite, and could walk three or four miles with no inconvenience, except some increase of the pain.

On instituting a physical examination I found the uterus retroverted, so as to permit the probe to enter freely when straight. The os was sufficiently patulous to admit the index to the first joint nearly, the anterior lip being somewhat enlarged on account of long standing retroversion. Posteriorly there was ulceration of the os and cervical canal of the granular variety, which bled from the slightest touch.

On account of this bleeding about the os, whenever the probe was introduced, and my inability to elicit tenderness of the organ, I failed to detect corporeal endometritis, until after the former parts were cured. Those of your readers who have made digital explorations of the internal genitals of fat women know that the accumulation of adipose tissue about the vulva places the uterus so much farther off that it greatly increases the difficulty and uncertainty of bimanual palpation. In this case I could not pass the finger farther than the cul-de-sac of Douglas. The fat on the abdomen increased the difficulty of diagnosis.

By the local application of caustics, astringents and alteratives, the inflammation and ulceration were cured after a long time, and the displacement was rectified and the uterus kept in position by a closed lever pessary.

When discharged she was entirely relieved of the pain and metrorrhagia, although she did not menstruate with entire regularity.

Perhaps it would be well here to give an account of a great, and perhaps unpardonable mistake that I made during the treatment of this case. It will surely reflect no credit on me, but perhaps it may be the means of saving some thoughtless and inexperinced brother from committing the same blunder, and I am sure if it had been some exploit of which I had reason to be proud I should be certain to tell it. In fact, I think we are bound to make our blunders public if the profession at large is probably to be benefited by our doing it.

After the disease of the os had been cured, and it had come down to the size of a goosequill, and when I had introduced the solid stick of argent. nit. into the uterine cavity several times, in order to avoid cauterizing the sound part every time; I introduced a goosequill, cut off at both ends, part of the way, and undertook to push the caustic through the quill, the latter protecting the part from the caustic, and to leave it (the caustic) in the uterine cavity, and strange to say the solid stick in the uterus caused little or no pain.

The plan succeeded admirably, until one day in passing the stick I accidentally pushed the quill entirely out of sight, into the organ.

I tried several expedients for extracting it, to no purpose, until the perspiration was pouring from my scared face, and there lay the poor lady, all unconscious of the danger she was incurring from the unskillfulness of her doctor. Finally an idea flashed into my affrighted mind, and taking a surgeons needle from my case, I turned the point on the sole of my boot, took it in the dressing forceps and delivered her of the goose-quill. I said nothing about it, and grandmother was too busy knitting to notice me.

